# Palliative care team visits. Qualitative study through participant observation 

**Published:** 2016-03-30

**Authors:** Maria del Mar Alfaya Góngora, Maria José Bueno Pernias, César Hueso Montoro, Plácido Guardia Mancilla, Rafael Montoya Juárez, Maria Paz García Caro

**Affiliations:** 1 Facultad de Enfermería de Melilla, Universidad de Granada, Granada, España; 2 Facultad de Ciencias de la Salud, Universidad de Granada, Granada, España; 3 Unidad de Cuidados Paliativos del Hospital Universitario San Cecilio de Granada, Granada, España

**Keywords:** Palliative care, home visits, ambulatory care, qualitative research

## Abstract

**Objectives:**

To describe the clinical encounters that occur when a palliative care team provides patient care and the features that influence these encounters and indicate whether they are favorable or unfavorable depending on the expectations and feelings of the various participants.

**Methods:**

A qualitative case study conducted via participant observation. A total of 12 observations of the meetings of palliative care teams with patients and families in different settings (home, hospital and consultation room) were performed. The visits were follow-up or first visits, either scheduled or on demand. Content analysis of the observation was performed.

**Results:**

The analysis showed the normal follow-up activity of the palliative care unit that was focused on controlling symptoms, sharing information and providing advice on therapeutic regimens and care. The environment appeared to condition the patients' expressions and the type of patient relationship. Favorable clinical encounter conditions included kindness and gratitude. Unfavorable conditions were deterioration caused by approaching death, unrealistic family objectives and limited resources.

**Conclusion:**

Home visits from basic palliative care teams play an important role in patient and family well-being. The visits seem to focus on controlling symptoms and are conditioned by available resources.

## Abstract (es)

**Objetivos:**

Conocer cómo se produce el encuentro clínico en la atención a los enfermos por parte de un equipo de Cuidados Paliativos, y qué elementos lo condicionan, indicando si son favorables o desfavorables en función de las expectativas y los sentimientos de los distintos participantes.

**Métodos:**

Estudio de casos cualitativo, realizado mediante observación participante. Se realizaron 12 observaciones de los encuentros de los equipos de cuidados paliativos con los pacientes y familiares en diferentes entornos (domicilio, hospital y consulta). El tipo de visitas fueron de seguimiento o primera visita, programadas o a demanda. Se realizó un análisis de contenido de las observaciones.

**Resultados:**

El análisis mostró una actividad normal de seguimiento de una unidad de cuidados paliativos centrada en el control de síntomas, la comunicación de información y el asesoramiento sobre pautas terapéuticas y cuidados. Se observó que los escenarios condicionan la expresión de los pacientes y el modo de relación. Como condiciones favorables del encuentro clínico destacan la afabilidad y la gratitud. Las condiciones desfavorables fueron el deterioro por la cercanía de la muerte, los objetivos poco realistas de los familiares y la escasez de recursos.

**Conclusión:**

Las visitas domiciliarias de equipos básicos en Cuidados Paliativos tienen un importante papel, y parecen estar centradas en el control de síntomas y condicionadas por los recursos disponibles.

## Introduction

Regarding factors that determine appropriate care at the end of life, most studies highlight access to palliative care, treatment objectives (curative vs palliative), the content of care (physical, psychological and spiritual) and the patient's and caregivers' participation in the process 1,2. 

Among the main causes of poor quality of care are the delay in establishing a diagnosis of terminal illness 3-6, limited discussion with the patient and family to agree upon and change treatment objectives 7,8, trends among professionals to provide information regarding unrealistic and often overvalued treatment options, and the difficulties faced by patients who wish to participate in decisions about the treatments they wish to receive at the end of life 8, 9-12.

Patients and families have identified good communication as a critical aspect of medical care at the end of life 1. Communication can be improved to enable patients to ask questions that concern them. Questions about sensitive topics, such as prognosis and other issues relating to the end of life, are known to be difficult to address with patients and families 1; however, such discussions are crucial to properly guide their expectations and make it easier for them to express their preferences 11. 

Similarly, professionals typically find it difficult to communicate with patients and families about the severity of a condition and to consider their options and preferences, especially if they differ from those of the professional 13,14. 

These difficulties arise in the context of the relationship that professionals have with patients and their families. Specifically, there are often face-to-face communication problems 15 and differing expectations in terms of information, communication and participation within the context of the medical consultation room 16. However, although the literature on end-of-life quality is broad, few studies have addressed the most immediate and direct level of care, that is, the clinical encounter, the relationship between the professional, the patient and the patient's family *in situ*. In this sense, qualitative methodologies, specifically participant observation or ethnographic study, have become very useful methodological tools.

The objectives of this study were to examine the form of clinical encounter with palliative care and the conditions under which it occurs and to determine the elements of palliative care that are favorable and unfavorable to the visit's objectives according to the expectations and feelings that emerge during the visit. 

## Materials and Methods

###  Design 

This was a qualitative case study using ethnographic participant observation and content analysis 17. According to the Polit and Hungler classification, case studies are in-depth investigations of a single entity or a reduced series of entities; the entity can be an individual, but can also include families, groups, institutions or other social units^18^. In this study, a case is defined as an observation. 

The palliative care team involved in the study is located in a city in southern Spain. It comprised a doctor and a nurse with three and two years of specific professional experience in palliative care, respectively. They are the only palliative care team in the town. They conducted home and hospital visits and attended consultations. 

A sampling strategy using logical criteria was used to select participants. The participants included both members of the palliative care team (the doctor and the nurse) and the patients and family members that they attended.

Given the possible context and conditions for the observations of selected cases, the following criteria were considered: a) the environment of the end-of-life visit-hospital, home or consultation room, and b) the reason for the visit-first visit or follow-up, requested or scheduled. All of the patients had advanced terminal cancer. 

Observations took place in all of the predicted scenarios (home, hospital room and consultation room) and for all reasons for consultation (first visit, follow-up, scheduled and requested). A total of 12 observations were made. Of the patients treated, 7 were men and 5 were women, and all were aged between 47 and 81 years. In all cases, one or more family members accompanied the patient. The cases are described in Table 1. 

**Table 1.** Description of observed cases.

Case	Age	Sex	Location*			Type of visit**				Family member
			D	H	C	1st	F	S	R		
**Ob-01**	65	F	x				x	x		Daughter
**Ob-02**	68	F		x			x	x		Daughter
**Ob-03**	62	M			x	x		x		Wife
**Ob-04**	52	M		x		x			x	-
**Ob-05**	72	M	x			x		x		Daughter
**Ob-06**	47	M	x				x	x		Sister-in-law
**Ob-07**	81	M	x				x	x		Wife
**Ob-08**	62	F			x	x		x		Son- and daughter-in-law
**Ob-09**	75	M	x				x	x		Daughter and wife
**Ob-10**	72	M	x				x	x		Wife and daughter
**Ob-11**	78	M	x						x	Wife
**Ob-12**	68	F	x					x	x	Daughter

*D: domicile, H: hospital, C: consultation room

**1st: first visit, F: follow-up S: scheduled, R: requested

###  Procedure

Data were collected using non-interfering systemic observation 18,19. The observed interaction occurred between the patients, their families and the professionals in the defined scenarios, which formed the organizing principle of the observations. The study was conducted in June and July 2014. 

In all cases, the observer was the investigator herself, and she accompanied the palliative team to the location of the clinical encounter. The investigator's role in field work is to participate as an observer without interfering or interacting in the clinical scenario in a manner that could be considered participation. The investigator was introduced in some cases as a professional who accompanied them that day; other times, she was introduced as a member of the team. 

The data collection instruments were the observation record and the field notebook. 

The observation record was organized around six items: previous contextualization (observation situation, relevant previous data, reason for visit); description of the observation scenario (type of scenario; sketches of the surroundings); non-verbal interaction (non-verbal communication of the participants, use of space); verbal interaction (the treatment dispensed, subject of conversation, language used, silences, specific questions, responses, emotional expressions); incidents (interruptions); and later contextualization (satisfaction/dissatisfaction, mood at the end of the interaction). 

### Data analysis

ATLAS.ti 6.2^®^ computer software was used for data analysis. The content analysis strategy was followed 18. First, we proceeded to organize the primary documents to create the hermeneutic unit. Primary documents were created using a dump of the information that was collected in the field notebook, and each observation in an identified case was represented by an alphanumeric code. After the allocation of the primary documents, a series of theoretical categories was established a priori according to the dimensions established in the observation record, which helped to organize the codes derived from the analysis of observations into families. 

Citations were identified in the primary documents and then coded, incorporating the codes that emerged from the analysis of the observation texts. The identified codes were grouped according to their characteristics under theoretical categories that were established *a priori*. The most complex categories were organized into subordinate concepts called sub-categories. The categories and sub-categories were the following: 

1. General scenario-Home setting and hospital setting 

2. Observation scenario-Home visit, hospital, consultation room, first visit, follow-up, on demand, scheduled 

3. Participants-Doctor, nurse, patient, family member/primary caregiver, observer, hospital team, other family members

4. Prior context-Information team (sharing information, preparing for visit); purpose of visit (implicit, explicit)

5. Expectations-Visit content (technical activities, information, support, advice); participants

6. Local scenario-Environmental characteristics; spatial distribution of the participants; home bedroom, hospital room, consultation room

7. Interaction-Verbal, non-verbal, subject, participants, incidents, general treatment, posture, physical expression, emotional expression, communication, feedback, questions, responses

8. Subsequent context-Effect of interaction, resolution of the visit, evaluation of the team, satisfaction, dissatisfaction, mood 

The cross-categories used for the segmentation analysis were the scenario, observation situation and participants. To determine favorable and unfavorable conditions, the previous context was taken into account, along with the expectations and subsequent context of the visits. 

The ethics committee authorized the project in general (PI-0670-2010) and specifically approved the field work by receiving authorization from the management of the hospital in which the palliative care team was located and the consent of the participating professionals.

Throughout the research process, any data that could identify the participants, professionals and patients was omitted; consequently, the name of the city in which the study was conducted was omitted because there is only one easily identifiable team in that city. 

To improve the study's reliability, data creation was controlled, as was the information recorded in the field notebook; the different types of notes (observation, methodologies, analysis and personal) collected in the observation record were distinguished. Mental and written notes were taken during the observations, and over the following 24 hours, an objective description of the observation was developed to distinguish the recorded data from the investigator's interpretations and reflections. The descriptions, analyses and results were subject to triangulation among researchers to ensure auditability and credibility. 

## Results

### Activities performed over the course of the clinical visit and the actions of the main actors (doctor, nurse, patient, family members) 

The observed visits were part of the palliative care unit's usual activity. The visits primarily took place in the patient's home and were scheduled; they were primarily follow-up and first visits. 

The descriptions of the observations regarding operation of the team highlight the strong role of the doctor, in contrast to the limited participation and absence of initiative on the part of the nurse, whose role was limited to helping and accompanying the doctor. 

In all cases, the patient took center stage when the action began. The patients generally responded to what was asked, recounting their symptoms and occasionally making comments about how they felt or what they thought of their situation:

"The patient openly shows their expectations of finding relief from his/her difficult situation with pain." (Ob 03)

*"The patient starts speaking very rapidly about his/her current state and detailing his/her main concerns. He/she appears very nervous and uneasy." *(Ob 07) *"The patient says he/she is tired of taking so many pills, that he/she knows they will not lead to a cure and prefers to be calm and at ease..." *(Ob 09)

Regarding the family members, there was always one (usually the wife or daughter) who served as the primary caregiver. The most common form of participation was providing information about the patient's daily life and reporting special circumstances. 

*"The main caregiver is her daughter... she takes us out to the hallway because she does not want her mother told that it is getting worse, saying that she will not be able to handle it." (Ob 02)* *"The caregiver is his wife, who has no knowledge of how to treat him at home, stating that he is too sick to be there. The patient, in turn, wants to be at home." *(Ob12)

Regarding the participants' activities and actions, the observations revealed the existence of routine activities that focused on symptom control as the main (sometimes only) topic. Other topics included information and advice about therapeutic guidelines and care and listen to and providing advice regarding the patients' and family members' concerns. 

*"The doctor begins the conversation with the patient asking how she is. (...) The doctor focuses on the symptoms that cause problems - pain and flatulence - and gives guidelines for better control." *(Ob 01)

*"The doctor changes some drug guidelines and increases the dose of others but above all, calms him..." *(Ob 03)

*"The nurse takes a blood pressure reading and records all data of interest for the patient's clinical history." *(Ob 05)

*"The nurse agrees with the patient about some periodic analyses that must be performed, and they agree on the day and time..." *(Ob 07)

The scenarios appeared to condition the patients' experience and the patient-professional relationship. In the home setting, the patients appeared more relaxed and generally exhibited more affective and intimate behavior. In scheduled follow-up visits, familiarity between the patients and professionals was noted. 

*"The patient specifically greets the doctor and nurse with two kisses, saying cheerfully that she is glad to see them." *(Ob 01). 

In the consultation room, interactions were more formal but still relaxed and comfortable, while in the hospital room, the interactions were more distant and concerned, and the consultation took the form of a typical doctor's visit. 

*"The visit was made at the request of the patient's family. The diagnosis has just been made, but the oncologist had not informed her of the poor prognosis. Her family wants her to know her prognosis. (...) The son knocks on the door of the consultation room and upon entering, greets with a 'Good morning, may we come in?'; then, he goes to the patient, greeting those present with a 'good morning' . The doctor was sitting, awaiting the arrival of the patient. A relaxed atmosphere is perceived... The patient is friendly and talkative, but clearly concerned..." *(Ob 08)

*"The visit is conducted in the hospital at the request of the oncology service. (...) The doctor entered first and greets, saying 'good morning', then moves on to the nurse and observer, who also greeted with this expression. The patient extends his hand to greet us upon entering, and his wife moved her head as a sign of greeting". *(Ob 04)

*"The visit was made at the request of the patient who, after having requested a voluntary discharge, was referred to the palliative care unit. He goes to the consultation room with his wife, so weak he comes in a wheelchair. (...) The patient is recounting to the doctor his experience during the last hospitalization; his wife is watching him, saying nothing. The patient spoke in a very low voice, speaking slowly, but expressing himself correctly. A relaxed atmosphere is perceived." *(Ob 03)

### Favorable and unfavorable conditions for achieving the objectives of the visit

**Figure 1.**
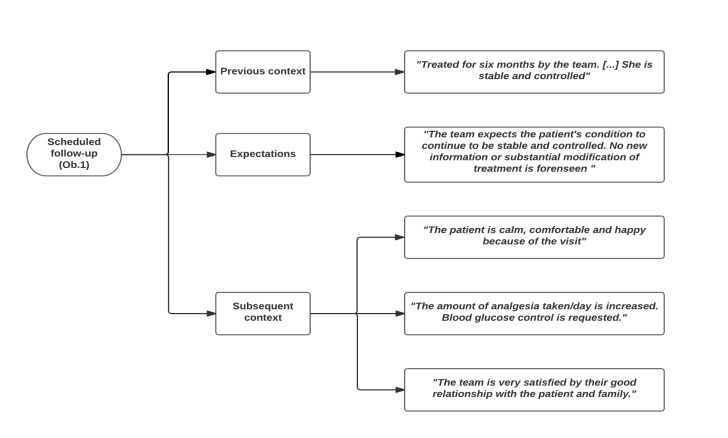
Situations that determine whether the visit objective is met: the previous context, expectations and subsequent context of home visits.

**Figure 2.**
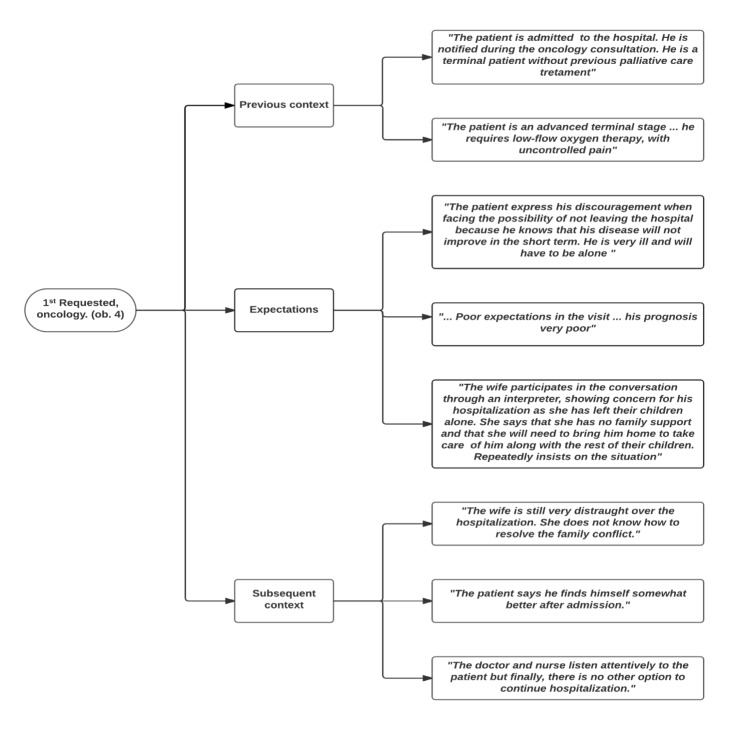
Situations that determine whether the visit objective is met: the previous context, expectations and subsequent context of hospital visits.

**Figure 3.**
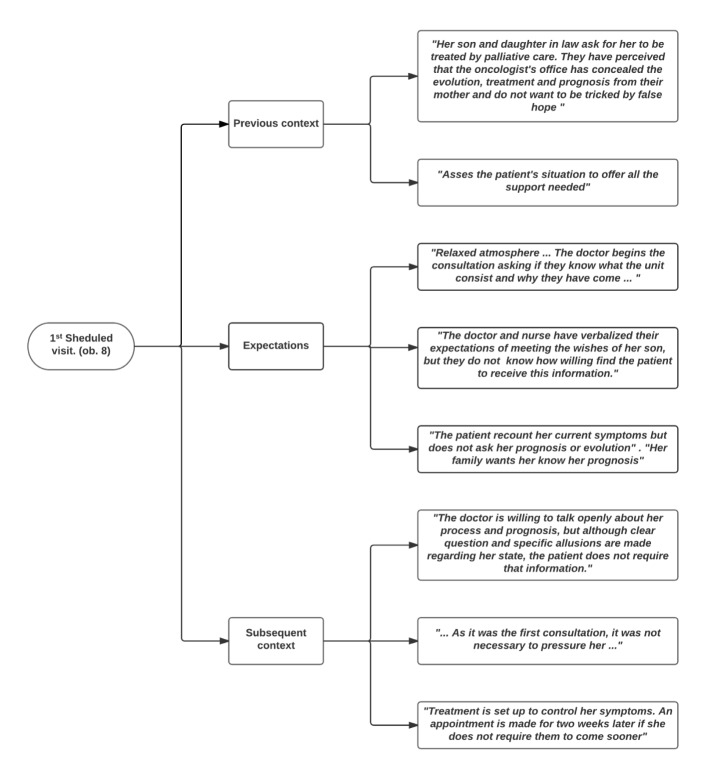
Situations that determine whether the visit objective is met: the previous context, expectations and subsequent context of consultation visits.

To determine the situations and features that promoted or hindered the objective of the visits, the previous and subsequent contexts and the expectations expressed or assumed by different actors were considered. Codes and quotes from different scenarios are shown in Figure 1 (home), Figure 2 (hospital) and Figure 3 (consultation room). 

The main purpose of this team's visits was follow-up and symptom control. Modifying specific problematic situations (within the social and psychological environment) was not a goal, neither was any other additional action.

The main expectations of the professionals in terms of follow-up focused on ensuring that the patient's symptoms and status were stable and managed. At first visits, the expectations focused on obtaining necessary information and ensuring the suitability of treatments to the patient's situation.

At requested visits, the expectations changed quite a bit depending on who made the request and where the visit took place. When the visit was requested by oncology for an admitted patient, the team's expectations ranged from concern about the patient's worsening state and uncertainty about the timing of death. When the request came from a family member, expectations were based on the reason for the request

The expectations of both the family members and the patients themselves were largely unknown to the professionals before the visit. The professionals assumed that concerns revolved around symptom control. However, on two occasions, family members explicitly voiced their expectations about the information the patient should or should not receive. During one visit, the caregiver expressed a desire to not communicate the poor prognosis of the disease to a sick relative. In another case, the family requested a visit in the palliative care consultation room to inform the patient about the poor prognosis of his/her disease

For the patients, interest focused primarily on symptom relief, discomfort and the particularly difficult situation of patients whose states were already well advanced

Among the conditions that could be considered favorable were the patients' friendliness during the palliative team's visit and expressions of gratitude at the end, regardless of the resolution of the problems that arose. In such cases, the information that the family and the patient had appeared to be adequate. There were no questions aimed at gaining additional information or efforts to receive a treatment or additional care. 

The conditions that were unfavorable because of their effect on both the patient's state and the interaction itself included the following: disease severity and physical deterioration caused by approaching death, unrealistic objectives and concerns among some relatives and a lack of resources to modify the conditions and situations of some patients. The visit routine also possibly reduced the initiative of the professionals to respond to complex problems when they arose. 

## Discussion

The objective of this study was to determine the manner and conditions under which the clinical encounter with palliative care patients occurred and to describe the elements that are favorable or unfavorable to such encounters based on the expectations and feelings they generate. Systematic observations of the daily activities that occurred during visits from a palliative care team were a useful method for determining the relevance of central aspects of the performed interventions. These observations revealed the difficulties of solving certain problems that the patients and families raised and the conditions under which the team operated. 

Regarding the activities that the palliative care team performed, symptom control was perceived as the most useful and effective, consistent with the published literature 20-22. Interventions focusing on psychological, social and family problems were very rare, as was the use of assessment tools that facilitate care in complex situations. However, these shortcomings did not impair the satisfaction of the patients and their family members. It is worth asking whether meeting the requests of the severely ill patient and treating them personally or following a set of visit guidelines represent favorable conditions that improve the state of the patient and their family, even when such approaches do not solve their problems. 

The therapeutic effect of doctor/nurse-patient 17 relationships is amply demonstrated in the scientific literature; however, it is worth asking whether such relationships, though necessary, are enough to meet the needs of terminally ill patients. Good intentions are not enough, and palliative care teams multidisciplinary by definition precisely because of the need for a comprehensive approach to caregiving at the end of life 20,22. 

In this sense, it should be noted whether the team structure and the relationship between the professionals in our study are appropriate for the development of this type of care. As noted in the technical literature 23, palliative care can be administered on at least two levels: the basic level (provided by teams of primary care doctors and nurses, general hospitals and nursing homes) and the specialized level (services whose primary activity is to provide palliative care by combining a multidisciplinary team with an interdisciplinary approach). Thus, whatever forms the human resources and level of care takes, in palliative care, the constant formula and standard of care is teamwork 24. The structure and the hierarchical relationship of the team observed in this study suggests a basic level of care; however, because the team is specifically designated as providing administering palliative care, it should be structured and operate at an advanced level. The absence of other professionals on the team is a significant limitation to providing a comprehensive response to the needs of patients and their families and could explain why the team's activity focused mainly on controlling symptoms. 

Moreover, regarding the relationship between the professionals, the nurse's lack of initiative both in providing specific information to the patient and caregiver and in care planning was surprising. Specifically, regarding the responsibility of the nurse, the reviewed literature refers to various types of activities and functions, including the responsibility for referring patients for palliative care 25, planning care with consideration for individual needs and continuity 26, serving as an expert in the physical assessment and evaluation of pain and other symptoms, directing care for the patient and family members and providing knowledge of the tools included in the care plan 27. However, other studies report barriers to achieving a good level of quality in such care 28. Among the difficulties noted are a lack of time, the work methods, professional autonomy 29, and a lack of training and specialization 30. Therefore, it is possible that the nurse's actions and the hierarchical relationship within the team could be explained by a lack of specialized training on the part of both the nurse and the doctor, as in Spain, this medical specialty is not yet been fully developed. 

In this sense, it is necessary to note the importance of strengthening communication channels to facilitate the transfer of information, collaboration and coordination among health professionals, which would have a positive impact on patient outcomes 31.

Finally, it should be asked whether the (geographic) location in which one dies is decisive in determining whether one type of care or another is provided. According to the literature on this topic 20, 32-35, it can be said that in Spain, differences in the development of palliative care among territories is an indicator of the existing inequality in end-of-life care. 

Considering the use of qualitative methodology and the fact that only a single palliative care team was evaluated, caution should be taken when generalizing this study's results to other contexts. However, a great number of different situations were analyzed, and the data analysis ended after theoretical saturation was reached. 

## Conclusion

The results of this study suggest that home visits from basic palliative care teams play an important role in patient and family well-being. However, the team configuration and the available resources can force care to focus mainly on controlling physical symptoms. 
